# Acute myeloid leukemia with rare recurring translocations—an overview of the entities included in the international consensus classification

**DOI:** 10.1007/s00277-024-05680-5

**Published:** 2024-03-06

**Authors:** Synne D. Rørvik, Synne Torkildsen, Øystein Bruserud, Tor Henrik Anderson Tvedt

**Affiliations:** 1 Department of Cardiology, Haukeland University Hospital, Bergen, Norway; 2 Department of Haematology, Oslo University Hospital, Rikshospitalet, Oslo Norway; 3 Acute Leukemia Research Group, Department of Clinical Science, University of Bergen, Bergen, Norway; 4 Section for Hematology, Department of Medicine, Haukeland University Hospital, Bergen, Norway

**Keywords:** Leukemia, Myeloid, Acute, Chromosome aberrations, Cytogenetics translocation, Genetic

## Abstract

Two different systems exist for subclassification of acute myeloid leukemia (AML); the World Health Organization (WHO) Classification and the International Consensus Classification (ICC) of myeloid malignancies. The two systems differ in their classification of AML defined by recurrent chromosomal abnormalities. One difference is that the ICC classification defines an AML subset that includes 12 different genetic abnormalities that occur in less than 4% of AML patients. These subtypes exhibit distinct clinical traits and are associated with treatment outcomes, but detailed description of these entities is not easily available and is not described in detail even in the ICC. We searched in the PubMed database to identify scientific publications describing AML patients with the recurrent chromosomal abnormalities/translocations included in this ICC defined patient subset. This patient subset includes AML with t(1;3)(p36.3;q21.3), t(3;5)(q25.3;q35.1), t(8;16)(p11.2;p13.3), t(1;22)(p13.3;q13.1), t(5;11)(q35.2;p15.4), t(11;12)(p15.4;p13.3) (involving NUP98), translocation involving NUP98 and other partner, t(7;12)(q36.3;p13.2), t(10;11)(p12.3;q14.2), t(16;21)(p11.2;q22.2), inv(16)(p13.3q24.3) and t(16;21)(q24.3;q22.1). In this updated review we describe the available information with regard to frequency, biological functions of the involved genes and the fusion proteins, morphology/immunophenotype, required diagnostic procedures, clinical characteristics (including age distribution) and prognostic impact for each of these 12 genetic abnormalities.

## Introduction

Acute myeloid leukemia (AML) is a heterogeneous condition characterized by genetic alterations that disrupt the differentiation of hematopoiesis and cause the expansion of immature myeloid blast cells. Approximately 50% of patients show chromosomal aberrations, with more than 100 different chromosomal aberrations described in AML. A subset of chromosomal abnormalities, termed recurrent genetic abnormalities, directly influence AML pathogenesis and are thus closely related to distinctive clinical features and survival. While AML classification was initially based on bone marrow blast cytomorphology, the presence of specific genetic aberrations is increasingly replacing traditional cytomorphology criteria.

Currently, two different classification systems exist for AML. The 5th edition of the World Health Organization Classification of Hematolymphoid Tumours (5th WHO-Hem) recognizes nine subtypes of AML defined by recurrent chromosomal abnormalities [1], and the International Consensus Classification (ICC) of myeloid malignancies recognizes 11 subtypes [2].

The ICC includes the subtype of AML with rare recurring translocations, which encompasses 12 different translocations that occur in less than 4% of patients with AML (Table 1). The data for these subtypes mainly come from small case series or aggregated data from large multicenter trials. Although these AML subtypes exhibit distinct clinical traits and are highly correlated with treatment outcomes, information on these entities is limited and often difficult to assess.

**Table 1:** The entities included in the international consensus classification

AML subtype	Chromosome location	Genes involved	Protein type/ function	Age group
AML with t(1;3)(p36.3;q21.3)	1p36.32	PRDM16	Zinc finger transcription factor and histone methyl-transferase activity.	
	3q21.3	RPN1	Ribophorin, an endoplasmic reticulum transmembrane protein.	
AML with t(3;5)(q25.3;q35.1)	3q25.32	MLF1	Nucleocytoplasmic shuttling protein	
	5q35.1	NPM1	Nucleolar protein, interacts with a wide variety of nuclear protein, involved in transport of nuclear proteins and organization of centromere.	
AML with t(8;16)(p11.2;p13.3)	8p11.21	KAT6A	Histone acetyltransferase and transcriptional regulator activities	
	16p13.3	CREBBP	Histone acetyltransferases	
AML with t(1;22)(p13.3;q13.1)	1p13.3	RBM15	Split-end family of proteins, repressor signaling pathways. RNA binding properties	Pediatric
	22q13.1	MKL1	Chromatin organization	
AML with t(5;11)(q35.2;p15.4)	11p15.4	NUP98	Nuclear pore complexes (NPCs) regulate the transport of macromolecules between the nucleus and cytoplasm	Pediatric
	5q35.2	NSD1	Transcription coregulator protein	
AML with t(11;12)(p15.4;p13.3)	11p15.4	NUP98	Nuclear pore complexes (NPCs) regulate the transport of macromolecules between the nucleus and cytoplasm	Pediatric
	12p13.33	KDM5A	Chromatin-regulation through histone demethylation and transcriptional repressor	
AML with NUP98 and other partner	11p15.4	NUP98	Nuclear pore complexes (NPCs) regulate the transport of macromolecules between the nucleus and cytoplasm	
AML with t(7;12)(q36.3;p13.2)	7q36.3	MNX1	Transcription factor.	Pediatric
	12p13.2	ETV6	Transcription factor.	
AML with t(10;11)(p12.3;q14.2)	10p12.31	MLLT10	Transcription factor and nuclear localization	
	11q14.2	PICALM	Regulates signals between several pathways (clathrin, phosphoinositols, receptor-mediated endocytosis).	
AML with t(16;21)(p11.2;q22.2)	16p11.2	FUS	RNA-binding protein	
	21q22.2	ERG(	Mitogenic signal transduction pathways and methylation regulation	
AML with inv(16)(p13.3q24.3)	16p13.3	GLIS2	Transcription factor, repressor of the Hedgehog signaling and Wnt signaling pathway.	Pediatric
	16q24.3	CBFA2T3	Transcriptional repressor	
AML with t(16;21)(q24.3;q22.1)	16q24.3	CBFA2T3	Transcriptional repressor	
	21q22.12	RUNX1	Transcription factor	

This review discusses the biological and clinical relevance of the ICC subtypes of AML with rare reoccurring translocations. The review also includes the total number of the specific mutations reported in the Mitelman Database of Chromosome Aberration and Gene Fusions in Cancer (last updated on the 3rd of August 2023). The study aims to describe its clinical characteristics and responses to treatment in order to provide a concise and easily available reference for clinicians.

### Acute myeloid leukemias with translocations involving NUP98 encoded on chromosome 11q15

#### Cytogenetic features and biological characteristics

Currently, more than 40 different translocations involving NUP98 have been described. While all AMLs with NUP98 translocations are defined as a single entity in the 5th edition of the WHO Classification, the ICC defines three different subtypes: (i) AML with NUP98:: NSD1 translocations, (ii) AML with NUP98::KMD5A translocations, and (iii) AML with NUP98 other translocation partners [1, 2]. While NUP98 translocations are seen in myelodysplastic syndrome (MDS), AML, blast-phase chronic myelogenous leukemia (CML), and t-ALL, NUP98 translocations are exceedingly rare in myeloproliferative disorders [3]. While some NUP98 translocations occur in multiple neoplasia, others are confined to specific hematological entities.

The NUP98 codes for nucleoporin 98kD protein. Nucleoporin 98kD protein is a member of the nuclear membrane complex that regulates protein and mRNA traffic between the cytoplasm and the nucleus [4]. NUP98 is found diffusely throughout the nucleus and is located in specific clusters associated with other transcription factors and chromatin-modifying proteins. All NUP98 translocation fusions are similar in that they involve the N-terminal portion of NUP98 and the C-terminal portion of the fusion partner [4, 5]. NUP98 is a type II mutation that leads to the impairment of hematopoietic stem cell differentiations through various mechanisms, including disruption of spindle formation, mitosis, aberrant DNA damage repair, and disruption of chromatin modulation [3, 4, 6]. A low mutational burden, gene expression profiles, and similar clinical characteristics indicate similar pathophysiological events in AML with NUP98 rearrangement [3, 4].

All NUP98 rearrangements detected in MDS are also seen in AML. Furthermore, the incidence of NUP98 rearrangements in the MDS population is extremely low, indicating rapid progression to AML after the initial genetic event [7].

### Clinical and molecular features

NUP98 rearrangements are typically cryptic and are associated with a normal karyotype [8]. The incidence of NUP98 rearrangements in AML is 3–5% in pediatric AML and approximately 1.3% in adult AML (Table 2), but it is 16.1% in pediatric AML and 2.3% in adult AML with a normal karyotype. To some extent, the observed difference in incidence in adult AML is explained by the practice of omitting NUP98 detection in adult AML.

**Table 2:** Studies on clinical characteristics and outcome in pediatric and adult patients with NUP98-NSD1 translocation

Study	Year	Pediatric /adult	Frequency	Sex Male/female	Median age (range)	FAB	Karyotype	Molecular	Survival
Hollink [5]	2011	Pediatric Adult	Pediatric 4.2% (293) Adult 1.3% (808)	65,2/ 24,8	16.8 (2.3 63.0)	M1:13% M2: 13% M4: 34,8 M5: 30,4%	78,3% normal	91,3% FLT3-ITD: 45% WT	Pediatric: CR 12/12 4-years CIR/EFS/ OS 83%/8% 31% Adult: CR 4/10 4-years CIR/EFS/OS 89%/32%/ 11%
Thol [7]	2013	Adult	7/504 (1.4%) AML MDS 0/193	3/4	34 (23–48)	M1/M2: 3/7 M4: 4/7	5/7 normal	FLT3-ITD 5/7 WT2/4 NPM1 0/7	CR 43% Approx. 25% long term survival
Strusk [12]	2017	Pediatric	22/574 3.8% NUP98 mutated 16/574 NUP98-NSD mutated	16/22	11.5 (3–17)	M1: 3/16 M2:3/16 M4:4/16 M4/M5; 1/16 M5: 5/16	9/16 normal 2/16 complex 5/16 trisomy 8	FLT3-ITD:13/16 WT: 3/16 CEBPA: 3/16	5-year EFS 30% 5-year OS 48%
Bolouri [11]	2018	Pediatric aAdult	Age 1–15 : 0,8% Adult 0,2%	NR	NR	NR	1/4 normal 1/4 del(9q) 1/4 abnormal 1/4 unknown	NR	CR 54.8%.
Marceau-Renaut [10]	2018	Pediatric	9/385 (2,3%)	NR	9.9 (1.3–16.8)	NR	5/9 normal	FLT3–ITD (7/10) WT1 (5/10), CEBPA 2/10 RUNX1 (2/10)	CR. 3 year-EFS 10% 3 year-EFS 25%
Shiba [14]	2019	Pediatric	11/369	5/1	7,2 (2–15)	M0,M1,M2: 3/11 M4:2/11 M5:6/11	5 normal 4 trisomy 8 1 complex 1 other	FLT3-ITD: 8/11	HR, 5.07(2.54–10.1) compared with other types
Niktoreh [13]	2019	Pediatric	15/246 (4%)	NR	NR	NR	NR	FLT3-ITD: 9/15 WT: 8/15	3-year CIR 81% 3 year-EFS 13% 3 year-EFS 52%
WU [119]	2023	Adult	NR	5/6	30 (14–59)	NR	9/11 normal 1/ trisomy 8 1 other	FLT3-ITD 7/11	CR 3/11 1-year OS 54.5%
Bertrums [15]	2023	Pediatric Adult	Pediatric: 108/ 2,235 Adult: 1.3% (13/825)	64.8% male versus 35.2% female patients	10.2	M6/M7 2,9%	18.8% trisomy 8 4,2% monosomy 5% del5q	FLT3-ITD 74% WT1 42%	CR 38.2% 5-year CIR 64% 5 year-EFS 17% 5 year-OS 36%

The Study by Osternoff [22] omitted sine some of the patients also were included in the study by Bertumset al [15]. Abbreviations: FAB: French–American–British classification, CR: Complete remission, NR not reported, CIR: cumulative incidence of relapse, EFS: event-free survival, OS: Overall survival

Approximately 75% of NUP98-mutated AMLs have the NUP98::NSD1 translocations [9]. The median age is 10 years for pediatric AML, with only a few cases reported in children younger than 2 years [5, 10–15]. Approximately 25% of NUP98-rearranged AMLs is therapy related [16]. AMLs with NUP98-NSD1 typically show M4/M5 morphology and a high leukocyte count [5, 7, 12, 15].

The karyotype in NUP98::NSD1 AML is usually normal. Other cytogenetic abnormalities occur in a small number of patients with trisomy 8; the deletion of chromosome 5 and a complex karyotype are the most commonly observed concurrent cytogenetic aberrations [5, 7, 14, 17]. FLT3-ITD mutations happen in most patients, with a small number also having WT1 mutations [13]. Screening for NUP98::NSD1 in adult patients with high leukocyte counts, normal karyotype AML, and FLT3-ITD mutations has been proposed.

While most other NUP98-mutated AMLs closely resemble AML with NUP98::NSD1, AML with NUP98::KMD5A (previously termed NUP98::JAR1A), NUP98::RARG, and NUP98::RARA show distinct clinical features [15].

Clinical and molecular characteristics of AML NUP98::KDM5A are summarized in Table 3. AML with NUP98::KDM5A typically presents as acute megakaryocytic leukemia (AMKL) (34%), at a significantly lower age (median: 3.2 years; range: 0 to 18) [16], with a lower white blood cell count, and has a very low frequency of WT1 and FLT3 mutations [12, 18, 19]. Structural chromosomal aberrations involving the RB1 gene are seen in more than half of patients [15].

**Table 3:** Studies on clinical characteristics and outcome in pediatric and adult patients with NUP98-KDM5a translocation

Study	Year	Pediatric/adult	Frequency	Sex (Male/Female)	Median age (range)	FAB	Additional chromosomal ab.	Molecular	Survival
Rooij [19]	2013	Pediatric 3 cohorts	4/293	1.6(1.2–5.9)	60%/40%	M5:1 M7:3	4/4 chromosomal rearrangements	NRAS 1/5	CR 78% 5-year CIR 56 5 year-OS 22
			11/105 pediatric AMKL	1.8(0.9–4.8)	45%/55%	M7:11	11/11 chromosomal rearrangements	KRAS 1/11	
Hara [18]	2017	Pediatric	4/44 AMKL	1 (1–2)	NR	M7: 4	1 Normal 3 complex	None	CR 3/4
Noort [120]	2021	Pediatric	47/2,393 (2.0%)	53/47	3,2 (0,07–18,5)	M0,M1, M2: 4/47 M4:2 M5:6 M6: 5 M7:10 Not know: 18	Cryptic rearrangement 29/44	FLT3/ITD 1/45 FLT3 point mutation* 1/29 CEBPA* 1/47 NPM1* 1/47 TET2* 0/25 ASXL1* 0/21 CKIT* 0/9 WT1* 3/29	CR 91% 5-year CIR 62,6% 5 year-EFS 29,6% 5 year-OS 34,1%
Bertrums [15]	2023	Pediatric Adult	Pediatric 2 Adult 0	18/14	2,7 (0.98–15.92)	M6/M7 N 46.9%	4 Trisomy 8 19 Chrom 13 ab	WT 1/32 FLT3 1/32	CR 80.6% 5-year CIR 68% 5 year-EFS 25% 5 year-OS 36%

Abbreviations: FAB: French–American–British classification, CR: Complete remission, NR not reported, CIR: cumulative incidence of relapse, EFS: event-free survival, OS: Overall survival

NUP98::NSD1 is usually positive for CD34 and CD117 and express the monocytic markers CD36 and CD64 in patients with concurrent FLT3-ITD. NUP98:KDM5A show a typical flowcytometric profile with CD34 negative blast cells and megakaryocytic maturation, partial expression of CD36 absence CD123 [15].

NUP98-RARG and NUP98-RARA have clinical phenotypes of acute promyelocytic leukemia with similar bone marrow morphologies, coagulation abnormalities, and immunophenotypes [20, 21]. Patients with NUP98-RARG seem resistant to all-trans-retinoic acid and arsenic trioxide treatment [20]. In vitro studies indicate that NUP98-RARA is sensitive to ATRA. No clinical data exist on the use of ATRA or arsenic trioxide treatment prior to complete remission [21].

### Response to chemotherapy and prognosis

NUP98::NSD translocation is associated with a poor prognosis, primarily because of the high rates of induction failure. The reported remission rates vary between 30% and 50%, the relapse rates between 50% and 80%, and the long-term survival between 30% and 50%; there is a five times higher risk of death compared with other non-NUP98::NDS1-mutated AML [7, 22]. Concurrent FLT3-ITD and WT1 mutations are associated with a dismal prognosis, with complete remission rates of 10% [13]. For patients with NUP98::KMD5A, several older studies reported low rates of complete remission [12, 23, 24], however a recently published study reported a complete remission rate of 80% [15]. Long-term survival remained poor because of high relapse rates [15].

### Acute myeloid leukemias with translocations involving transcriptional regulators encoded on chromosome 16

#### Cytogenetic features and biological characteristics

This subset includes three translocations involving chromosome 16: CBFA2T3::GLIS2, t(16;21)(q24/q22)/ RUNX1::CBFA2T3, and t(16;21)(p11;q22)/ FUS::ERG. All three translocations involve genes that encode proteins involved in transcriptional regulation. The CBFA2T3::GLIS2 translocation seems to be a pediatric variant [25, 26], whereas the two other variants are also detected in adults [27, 28]. The leukemia cell morphology shows a wide variation for all three variants. Although some cytomorphological variants show increased frequencies for some of them (e.g., AMKL is more common for the CBFA2T3::GLIS2 variant), it is important to emphasize that such variations are not absolute [25, 27, 28]. Furthermore, the CBFA2T3::GLIS2 variant cannot be diagnosed by karyotyping, but can be suspected by immunophenotyping [29]. The two other translocations can be detected by karyotyping.

### Clinical and molecular features

The three translocations are all uncommon. They involve genes that are important in transcriptional regulation; new fusion genes/proteins are thereby formed by the translocations, and these fusion proteins alter transcription regulation and contribute to malignant transformation. The CBFA2T3::GLIS2 translocation can occur in patients with a normal karyotype [26], and the two t(16;21) translocations can occur as the only cytogenetic abnormality for a small number of patients [27, 30]. All three anomalies can be detected in combination with a variety of cytogenetic and molecular genetic abnormalities, and it is difficult to know on the basis of the available data how the different genetic abnormalities interact in the process of malignant transformation. The characteristics of each of the three variants are described in detail in Table 4.

**Table 4:** AML with translocations involving the CBFA2T3 gene (synonyms ETO2; MTG16; MTGR2; ZMYND4; RUNX1T3; see the Gene database). The full official name of this gene is CBFA2/RUNX1 partner transcriptional co-repressor 3, and it encodes a member of the myeloid translocation gene family which interacts with DNA-bound transcription factors and recruit a range of corepressors to facilitate transcriptional repression (information from the Gene database, accessed 230,213)

	CBFA2T3-GLIS2 translocation	t(16;21) (q24/q22)/ RUNX1-CBFA2T3	t(16;21)(p11;q22)/ FUS-ERG
Frequency	It is the most frequent oncogene identified in pediatric non-Down Syndrome acute megakaryoblastic leukemia, being detected in 15–20% of these cases [25, 26].	An infrequent but recurrent AML variant. Different RUNX1 translocations have been described in various hematological malignancies, the RUNX1T3/CBFA2T3 fusion being the fifth most frequent partner in cancer-associated RUNX1 rearrangements [121].	This is an uncommon AML variant both in in children and adults [27]. It is a recurrent translocation in acute leukemia and in certain solid tumors [28].
Chromosomal abnormality	A cryptic inversion of chromosome 16.the lesion can thus not be identified by cytogenetic analysis [122]. Concomitant cytogenetic abnormalities are uncommon; about 30% of these patients have a normal karyotype and the other patients can have various cytogenetic abnormalities including complex karyotype and hyperdiploidy [24].	The translocation involves the RUNX1T3/CBFA2T3 gene on chromosome 16q24 and the RUNX1 gene on chromosome 21q22. The oncogenic fusion protein has a structural similarity with the oncogenic protein RUNX1-RUNX1T1 [30].	This t(16;21) abnormality is the sole cytogenetic abnormality in 30–40% of patients [27].
Additional genetic abnormalities	Patients with this lesion have a lower mutational burden than other AML patients. Combinations with *FLT3, GATA1, KIT, RAS* and *JAK/STAT* mutations have been described [24, 25, 103].	The translocation can be the only cytogenetic marker, but a majority of the patients have additional cytogenetic abnormalities [30]. It can be detected at the first time of diagnosis or develop during disease progression (CML blast phase) or at the time of relapse [30]. Various molecular genetic abnormalities have been detected in combination with this translocation, the translocation can be a part of a complex karyotype or be observed in patients with clonal cytogenetic heterogeneity [30]. The most frequent additional abnormalities are trisomy 8 and del(7q) [30].	The abnormality can be detected especially in combination with trisomy 8 and trisomy 10; a complex karyotype is seen for one third of the patients [27]. Several abnormaities in genes encoding epigenetic regulators have been detected in combination with the translocation (e.g. DNMT3A, ASXL1, BCOR) [28]
Function of the translocated gene	CBFA2T3. The full official name of this gene is CBFA2/RUNX1 partner transcriptional co-repressor 3, and it encodes a member of the myeloid translocation gene family which interacts with DNA-bound transcription factors and recruit a range of corepressors to facilitate transcriptional repression (Gene database). GLIS2: The encoded molecule is a member of the Krüppel-like zinc finger transcription factor group; animal studies suggest it be involved in hematopoietic stem cell regulation but it is not expressed in differentiating hematopoietic cells. Fusion protein: The fusion protein does not include the CBFA2T3 molecular domain involved in binding to the nuclear receptor-coreceptor complex. The zinc finger domain of GLIS2 and thereby the ability to bind to DNA is maintained in the fusion protein.	RUNX1 (Runt-related transcription factor 1): The encoded protein is a transcription factor. Core binding factor (CBF) is a heterodimeric transcription factor that binds to the core element of many enhancers and promoters of transcription. The protein encoded by this gene represents the alpha subunit of CBF and is involved in the development of normal hematopoiesis (Gene database). Fusion protein: The RUNX1-CBFA2T3 fusion protein functions as an altered transcriptional corepressor able to recruit histone deacetylases and thereby capable of suppressing the expression of RUNX1 target genes	FUS. The official name is FUS RNA binding protein. This encoded multifunctional protein is a component of the heterogeneous nuclear ribonucleoprotein complex. This complex is involved in pre-mRNA splicing and the export of fully processed mRNA to the cytoplasm. This protein belongs to the FET family of RNA-binding proteins which have been implicated in cellular processes that include regulation of gene expression, maintenance of genomic integrity and mRNA/microRNA processing (Gene database). ERG: The official name is ETS transcription factor ERG. The encoded protein is a member of the erythroblast transformation-specific (ETS) family of transcriptions factors. All members of this family are key regulators of embryonic development, cell proliferation, differentiation, angiogenesis, inflammation, and apoptosis. The protein is mainly expressed in the nucleus. It contains a DNA-binding domain and a PNT (pointed) domain which is implicated in the self-association of chimeric oncoproteins; it is also a regulator of hematopoiesis including differentiation and maturation of megakaryocytic cells. Fusion protein. Different fusion variants of FUS-ERG have been described [28].
Clinical characteristics	Detected in children, most patients being younger than 5 years of age [25, 123]. The degree of peripheral blood leukocytosis is comparable to other AML patients, but they seem to have relatively high bone marrow blast counts. Extramedullary involvement is more freuwnt (25% of patients) than for toehr pediatric AML patients [25].	The translocation is observed both in children and adult patients [28]. The majority of patients have *de novo* AML, but it can also be secondary to previous chemotherapy (e.g. sarcoma treatment including alkylators and topoisomerase 2 inhibitors with a median latency of 24 months (range 12–108 months [30]. It has also been described in patients with CML-blast phase [30]]. Therapy-related forms are observed both in children and adults [30]. Circulating blast levels are usually relatively low (< 20 × 10^9^/L), levels > 100 × 10^9^/L are uncommon [27].	Detected both in children and adult patients. It can be secondary to previous chemotherapy [27]. Circulating blast levels are usually relatively low (< 20 × 10^9^/L), levels > 100 × 10^9^/L are uncommon [27].
Morphology	The abnormality is common in patients with acute megakaryoblastic leukemia, but apptoximately half of the patients seem to have a non-FAB M7 phenotyp that can be both M0, M1, M2 and M4/M5 [122].	The morphology shows relatively large blasts with both a prominent nucleolus and perinuclear hof; dysplastic granulopoiesis can be seen whereas dysplastic erythropoiesis/megakaryopoiesis is uncommon [30]. Eosinophilia can be a part of the morphological picture [124]. Most of the patients have a FAB-M1/M2 morphological phenotype [27]].	The abnormality has no predominant FAB type. Eosinophilia, micromegakaryocytes and hemophagocytosis can be a part of the microscopic picture [27]. Basophilic leukemia has also been described [125].
Immunophenotype	The typical phenotype is overexpression of CD56 and low expression of CD38 (dim to negative) and HLA-DR. The diagnosis can thus be suspected by flow cytometry. another characteristic is dim-to-negative CD45 expression [29].	Blasts are often positive for the myeloid markers CD13 and CD33, the stem cell markers CD34 and CD117, CD38, HLA-DR and myeloperoxidase; abberant CD19 expression can be seen whereas they are usually negative for aberrant expression of T cell markers [30].	The AML blasts cab express CD11b, CD13, CD18, CD33, CD38, CD56 and CD117; myeloperoxidase can be negative [126, 127]. HLA-DR expression is variable [127].
Clinical outcome	Overall survival rate of 15–30% [66, 103].	A majority of patients seem to reach complete hematological remission after intensive induction chemotherapy, but despite this a median overall survival of only 22 months has been reported [30]. Results from a recent pediatric study (23 patients, five with secondary AML) showed a four-years event-free survival of 77% [27].	Complete remission is usually obtained (> 80% of patients) after intensive induction chemotherapy, but relapse is common and the 4-year overall survival in a pediatric group of patients was < 10% [27].

### Response to chemotherapy and prognosis

The possible prognostic impact of these three genetic abnormalities has only been investigated in small patient populations. Some conclusions are suggested by the available data, although the observations should be interpreted with great care. First, the CBFA2T3::GLIS2 translocation seems to be associated with an adverse outcome, and many studies suggest an overall four- to five-year survival rate of less than 30% [26]. Taken together, these studies suggest that survival is lower than that of fusion-negative patients and that many of these patients present with resistant diseases. Second, a pediatric study suggested that the RUNX1::CBFA2T3 variant has a more favorable prognosis, at least in children, with a four-year overall survival rate of 74% [27]. However, whether this also applies to adult patients, especially adults with secondary AML, is currently unclear. Finally, the FUS::ERG variant is regarded as a high-risk abnormality. Although most patients seem to achieve complete hematological remission after intensive chemotherapy (< 80% of pediatric patients), they have a high risk of later relapse, and in pediatric patients, the relapse risk seems to exceed 70% [27]. Finally, both t(16;21) translocations seem to be independent risk factors, at least for pediatric patients [27].

### AML with t(8:16)(p11.2;p13.3)/MYST3::CREBBP

#### Cytogenetic features and biological characteristics

The t(8;16)(p11;p13) translocation leads to the fusion of MYST3 on chromosome 8p11 and of CREBBP on chromosome 16p13, leading to an MYST3::CREBBP chimeric protein. MYST3, previously termed KAT6A, has protein with zinc-finger and acetyltransferase domains and acts as a co-activator transcription factor regulating hematopoiesis, such as RUNX1, PU.1, and NF-KB [31–33], while CREBBP is a co-activator of hematopoietic transcription factors that regulate hematopoietic stem cell stemness [34–37]. MYST3::CREBBP chimera protein is thought to disrupt hematopoiesis through aberrant chromatin acetylation and interaction with transcription factors [38]. AML with t(8;16)/MYST3::CREBBP has been shown to share many clinicopathological features with AML with t(11q23;v)/KMT2A rearrangements. Both entities are associated with infant AML or t-AML, extramedullary disease, and monocytic/monoblastic or myelomonocytic differentiation, and they may be related to the important roles that the MYST3::CREBBP and KMT2A fusion genes play in histone modification [8]. A total of 159 cases of AML carrying the t(8;16)(p11;p13) translocation are currently registered in the Mitelman Database of Chromosome Aberration and Gene Fusions.

### Clinical and molecular features

AML with t(8;16)(p11;p13) accounts for 0.2–0.4% of all AMLs and 1.6% of therapy-related AMLs. It can occur at all ages, with a peak during infancy and between 52 and 60 years. Pediatric cases are usually *de novo*, and for adults, they are often therapy related [8, 35, 39, 40].

The typical features of AML with t(8;16)(p11;p13) are extramedullary disease, intravascular coagulation, monocytic/myelomonocytic differentiation, and erythrophagocytosis. The characteristic flow cytometric findings are a bright CD45 expression and high side scatter, making it difficult to distinguish blasts from maturing myeloid elements. Blasts typically express CD13, CD33, and CD64 and are negative for CD34 and CD117 [40, 41]. Patients with therapy-related cases often exhibit a complex karyotype, while t(8;16)(p11;p13) is typically the sole genetic abnormality in patients with de novo AML [42]. Common secondary aberrations include trisomy 8 and trisomy 21. FLT3-TKD is frequent [43].

### Response to chemotherapy and prognosis

Although a complete response is achieved in 80% of patients, most relapse within the first year [35, 40, 43]. The factors associated with inferior survival are antecedent hematological malignancies, therapy-related AML, and a complex karyotype [43]. The MYST3::CREBBP transcript can be used to assess minimal residual disease [44]. However, the significance of MYST3::CREBBP molecular minimal residual disease before and after allogeneic stem cell transplant-HCST is not known [43].

Interestingly, a significant number of spontaneous remissions have been described in pediatric patients and in one adult individual [44]. However, the relapse rates are high following spontaneous remission, thus requiring long-term monitoring.

### AML with t(10;11)(p12-13;q14-21)/PICALM::MLLT10

#### Cytogenetic features and biological characteristics

The t(10;11)(p12-13;q14-21) translocation leads to the formation of a PICALM::MLLT10 fusion gene previously designated as CALM::AF10. PICALM, the phosphatidylinositol binding clathrin assembly protein gene, located on chromosome 11, encodes a protein involved in endocytosis that also co-locates to the nucleus, while the MLLT10 gene located on chromosome 10 encodes a nuclear protein within the DOT1L protein [45–47]. It is believed that the fusion transcripts disrupt MLLT10 functions so that DOT1L is misdirected to the promoters of certain HOXA genes that encode the transcription factors involved in hematopoiesis, leading to the hypermethylation of H3K79 and preventing cell maturation and differentiation [48–50]. PICALM::MLLT10 translocation occurs in AML, ALL, and acute undifferentiated leukemia. Because of the spatial proximity of the PICALM gene to KMT2A, PICALM::MLLT10 can be misinterpreted as the t(10;11)(p11-13);q23 translocation; thus, fluorescence in situ hybridization (FISH) or molecular analysis is required for accurate identification [45]. While PILCAM::MLL occurs in 10% of adult and pediatric T-ALL cases [45, 46], less than 100 cases of PICALM::MLLT10 have been reported in AML [45, 51, 52].

### Clinical and molecular features

Fewer than 100 patients with PICALM-MLLT10-mutated AML have been reported [45, 51, 52]. In the Mitelman Database of Chromosome Aberration and Gene Fusions in Cancer, there are now 79 cases of AML carrying the t(10;11)(p12-13;q14-21) translocation. This translocation occurs at all ages, mainly in late adolescence, with a median age of 20 years. PICALM::MLLT10-mutated AML occurs as both *de novo* and secondary AML [45, 53]. Because of the low number of patients reported, drawing clear conclusions on clinical and laboratory characteristics is difficult. However, extramedullary leukemia seems common, with a report on central nervous system and extensive skin involvement [54]. PICALM::MLLT10 AMLs typically exhibit immature cytomorphology and express CD13, CD33, CD34, CD65, CD117, HLA-DR, myeloperoxidase (MPO), and the T-cell antigen CD7; in some cases, they have mixed lineage phenotypes [16, 53].

Mark et al. reported the outcome of 39 individual’s age ≤ 21 with PICALM-MLLT10-mutated AML. The disease only occurred in older children with a median age of 14 with no cases younger than 9 or older than 15 years. The majority of cases showed either minimally differentiated (FAB M0/M1 16/39) or monocytic differentiated (FAB M5 7/39). CNS and extramedullary leukemia was only reported in 1 and 2 respectively [50].

In 50% of cases with AML and t(10;11)(p12-13;q14-21), no other cytogenetic abnormalities are found. The most frequently observed secondary changes include trisomy 4, trisomy 19, and deletion of 17p [55]. While rearrangements of the immunoglobulin heavy chain and T-cell receptor genes are frequent [46, 55–57], data on other concurrent mutations are scarce. Grossman et al. presented a case with a concurrent EZH2 mutation and speculated that EZH2 mutations and PICALM::MLLT10 are related because of possible synergistic effects on the deregulation of HOX gene expression [58]. Mark et al. reported RAS pathway mutation 21%, WT1 mutations in 12%, NOTCH in 6% and EVT6 mutations in 3% of patients [50].

### Response to chemotherapy and prognosis

The prognostic impact of PICALM::MLLT10 in AML is not well defined. Although an in vitro model suggested resistance to conventional chemotherapeutic treatment, this is not supported by observational data [59]. In a study of 18 patients by Borel et al., the complete response rates were 71%, and they did not differ from those of PICALM::MLLT10-negative AML. However, the relapse rates were high, with a 50% survival rate at 12 months [55]. In pediatric cohort reported by Mark et al. the 5-year event-free an overall survival was 22% and 26% respectively, with relapse being the most common cause of death [50]. Interestingly, long-term survival was observed after consolidation with both allogeneic stem cell transplant and high-dose cytarabine, but most patients were treated with the former. Pharmacological inhibition of the histone methyltransferase DOT1L has been suggested as a potential target in leukemias with PICALM::MLLT10 [60].

### AML with t(1;22)(p13.3;q13.1)/RBM15::MKL1

#### Cytogenetic features and biological characteristics

The t(1;22)(p13.3;q13.1) translocations result in a fusion of the oncogene RNA-binding motif protein-15 (RBM15) on chromosome 1 and of megakaryocytic leukemia-1 (MKL1) on chromosome 22. The fusion results in the relocation of the MKL1 nucleus and the constitutive activation of downstream pathways [61]. Transgenic mice with t(1;22)(p13.3;q13.1) show abnormal hematopoiesis and aberrant expression of cytokines, but transformation to AMKL occurs only in a fraction of transgenic mice [62]. Additional immunogenic or mutational events are required for leukemic transformation, but additional genetic events have not yet been identified.

### Clinical and molecular features

AML with t(1;22)(p13.3;q13.1)/RBM15::MKL1 almost exclusively occurs in children [61, 63], and only a few adult cases have been reported. AML with t(1;22)(p13.3;q13.1)/RBM15::MKL1 presents as AMKL and accounts for 50% of non-Down-syndrome AMKL [64]. With a peak incidence at 6 months and with most patients being below 3 years of age, the time of presentation is significantly lower than that of other AMKL types. There is a female preponderance. In the Mitelman Database of Chromosome Aberration and Gene Fusions in Cancer, there are now 65 cases of AML carrying this translocation.

Only two case reports of adult AML carrying t(1;22)(p13q13) have been reported. Saito et al. reported on a patient with AML following four months of treatment for a non-mediastinal germ cell tumor. After receiving a conventional 7 + 3 induction regimen, the patient achieved complete remission and received three cycles of high-dose cytarabine before allo-HSCT [64]. Although the patient experienced severe acute graft-versus-host disease, he remained in remission more than 200 days after transplantation. Hsiao et al. reported a 59-year-old male with AML with 46 XY, +der(1)t(1;22)(p13,q13) [65]. The patient achieved remission after 3 + 7 induction therapy, one course of 2 days of anthracycline and 5 days of cytarabine, followed by four courses of a high dose of cytarabine. At one year, the patient was in complete remission but with persistence of the fusion transcript, and he relapsed at 18 months. A second complete remission was achieved after salvage chemotherapy with mitoxantrone and etoposide, but the patient relapsed and died within 3 months.

Most cases have t(1;22)(p13.3;q13.1) as the sole karyotype abnormality at diagnosis [63]. A hyperdiploid karyotype with t(1;22) and + der(1)t(1;22) is seen in a small number of patients [63, 66–68]. With the exception of small case series describing the absence of FLT3-ITD, WT, and nucleolar phosphoprotein nucleophosmin 1 (NPM1), data on the mutational landscape in AML with t(1;22)(p13.3;q13.1)/RBM15::MKL1 are scarce [63].

Most patients show pancytopenia with normal or elevated platelet counts [67]. Clinical characteristics at diagnosis include pancytopenia and significant hepatosplenomegaly that sometimes impair venous abdominal drainage [61, 67, 68]. A significant number of cases present as extramedullary disease, with less than 20% of blast cells in the blood or bone marrow. Marrow aspiration is often difficult because of extensive marrow fibrosis. Because of extensive extramedullary disease, bone marrow fibrosis, and cytomorphological small round blue cells, AML can initially be misinterpreted as medulloblastoma, hepatoblastoma, or Ewing sarcoma [69]. Blast cells typically express CD31, CD41, CD42b, NSE, factor VIII, and CD61, and they have variable positivity for MPO [70]. In the Mitelman Database of Chromosome Aberration and Gene Fusions in Cancer, there are now 65 cases of AML carrying this translocation.

### Response to chemotherapy and prognosis

The outcome of pediatric AMKL is regarded as favorable, with a long-term survival rate of approximately 70%. Some studies have reported high early death rates in patients with extensive abdominal extramedullary disease at diagnosis. However, in contrast to most other non-Down syndrome AMKLs, AML t(1;22)(p13q13) has a high response rate after intensive chemotherapy and a favorable prognosis. Allogeneic stem cell transplantation is not recommended in the first remission.

### AML with t(3;5)(q25.3;q35.1)/NPM1::MLF1

#### Cytogenetic features and biological characteristics

The translocation t(3;5) leads to the formation of the NPM1::MLF1 chimeric gene involving NPM1 on chromosome 3 and of the myeloid/myelodysplastic leukemia factor 1 (MLF1) gene on chromosome 5. The translocation has only been described in AML or MDS [71]. While normal hematopoietic tissue does not express MLF1, approximately a quarter of high-risk myelodysplastic syndrome and MDS-associated AML show overexpression of MLF1. It is believed that the NPM1::MLF1 fusion induces leukemogenesis by promoting ectopic MLF1 expression in hematopoietic cells. NPM1::MLF1 and AML with NPM1-positive AML share similar flow cytometry and gene expression profiles and thus probably similar leukemogenic events [72].

### Clinical and molecular features

The t(3;5)(q25;q35) NPM1::MLF1 fusion happens in 0.5% of AMLs and occurs at all ages, with a reported median age between 24 and 47 years and with 18.4% being older than 60 years [51, 71, 73]. Young patients are frequently male, while older patients are typically female. The common bone marrow findings are three-lineage dysplasia and blast cell with myeloid maturation typically characterized as FAB M2. The flow cytometric profile is similar to that of NPM1-mutated AML, with blast cells being negative for CD34 and positive for CD117, CD13, and CD33 [71]. In younger patients, this translocation is usually the sole karyotypic abnormality, whereas older patients often exhibit a complex karyotype [71]. In the Mitelman Database of Chromosome Aberration and Gene Fusions in Cancer, there are now 8 cases of AML and 2 cases of MDS carrying this translocation.

### Response to chemotherapy and prognosis

Although the complete remission rate after intensive chemotherapy is high, most patients relapse within the first year [74], and the long-term survival rate is poor. One study reported a long-term survival rate of 34% at 10 years [51].

### AML with t(7;12)(q36.3;p13.2)/ETV6::MNX1

#### Cytogenetic features and biological characteristics

The translocation t(7;12)(q36;p13) involves the MNX1 and ETV6 genes. Translocation occurs in both AML and ALL. AML with t(7;12)(q36;p13) exhibits several distinctive characteristics [75]. First, the fusion transcript is only detected in half of the patients, a corresponding MNX1::ETV6 protein is not identified, and the leukemogenic effects of the fusion transcript are questionable [76–78]. Second, the entire MNX1 gene, including regulatory domains, is translocated to chromosome 12. This results in the overexpression of MNX1 [79]. Experimental models show that ectopic MNX1 expression mediates the leukemogenic effect through a blockage in the differentiation of hematopoetic stem cells and aberrant methylation that results in histone modifications, accumulation of DNA damage, and leukemia transformation [79]. Third, AML with t(7;12)(q36;p13) is highly age specific, with all cases restricted to children below 2 years of age. One possible explanation for the incapability of MNX1 ectopic expression in adult progenitor cells is dramatic apoptotic induction through p53/p21-dependent cell cycle arrest, which has not been observed in hematopoietic progenitor cells of fetal origin [77]. Lastly, animals transduced to express MNX1 only show leukemic transformation in immunocompromised recipients, supporting the assumption that specific immunological events in the developing immune system of newborns facilitate the disease [80].

### Clinical and molecular features

AML with t(7;12)(q36;p13) is mostly restricted to infants (defined as 0–2 years old), with a peak at 6 months [75, 81], and accounts for 18–30% of AML in infants, thereby being the most common cytogenetic abnormality in this age group. The prevalence could be somewhat underestimated, as the translocated regions are subtelomeric and not related to specific bands and are therefore difficult to detect with conventional karyotyping [75].

AML with t(7;12)(q36;p13) often show additional copies of chromosomes 8, 19, and 22 and screening for cryptic t(7;12) in young children with trisomy 19 is recommended [75, 82]. Thrombocytosis is common, and blasts are typically poorly differentiated, usually categorized as FAB M0, M1, and M2. However, several cases with M7 morphology have been described [81]. Blasts typically express CD34, CD117, HLA-DR, CD4, and CD7 [76, 83]. In the Mitelman Database of Chromosome Aberration and Gene Fusions in Cancer, there are now 29 cases of AML carrying this translocation.

### Response to chemotherapy and prognosis

AML with t(7;12)(q36;p13) is associated with poor clinical outcomes [76]. However, more recent survival analyses by Espersen et al. reported improved prognostic outcomes with a three-year event-free survival rate of 43% and a three-year overall survival rate of 100% [82] (Table 5).

**Table 5:** Studies on AML with t(7;12)(q36.3;p13.2)/*ETV6*::MNX1*

Study	Year	Pediatric /adult	Frequency	Sex (Male/Femal)	Median age (range)	FAB (number)	Chromosomal	Molecular	Survival
Espersen [82]	2018	7/0	7/651 (1.1%)	1/6	6 (2–8 mo)	M0: 1/ M1: 3 M2: 1/ M7: 1 Unclassified (1)	t(7;12)(q36;p13) + 19		3-year EFS 43% 5-year EFS 43% 3-year OS 100% 5-year OS 83% Relapse 57%
Espersen [76, 82]	2017	35/0	35	16/19	6 (2–24 mo)	M0: 8/ M1: 4 M2: 5/ M4: 2 M5: 3/ M7: 3 RAEB-T: 1/ MPAL: 2 Unclassified: 7	t(7;12) 30/35 had + 19		Outcome available in 22/35 3-year EFS 24% 3-year OS 42% Relapse 57%
Park [76]	2009	3/0	3/215 (1,4%)	3/0	7 (3–12 mo)	M0: 1 M5a: 1 Biphenotypic leukemia: 1	t(7;12) t(5;7;12) t(1;7;12) additional chromosome 19 in all cases	HLXB9 overexpression In all cases	2 patients dead in relapse 1 patients early death during induction
Slater [81]	2001	10	10/130 (7,7%)	4/6	6 (4–18 mo)	M0: 1/ M1: 2 M1/ M2: 2 M3v: 1/M4:1 M5: 1/ M7: 1 RAEB-t (1)	t(7;12)		All dead within 56 months

Abbreviations: FAB: French–American–British classification, CR: Complete remission, NR not reported, CIR: cumulative incidence of relapse, EFS: event-free survival, OS: Overall survival

### AML with t(1;3)(p36;q21)

#### Cytogenetic features and biological characteristics

Chromosomal band 1p36 is a recurring breakpoint involved in a variety of rearrangements in hematological neoplasms, with the most frequent being t(1;3)(p36;q21), and it has been reported in AML, MDS, CMML, CML, and ALL [84–86]. The t(1;3)(p36;q21) translocation involves the MEL1 (PRDM16) gene at 1p36.3 and the RPN1 gene at 3q21. The breakpoints for the RPN1 gene are located within a 60 kb region centromeric to the breakpoint cluster region of the 3q21q26/inv(3) involved in MECOM-rearranged AML [87, 88]. The t(1;3) results in the transcriptional upregulation of the MEL1 gene through promoter swapping with the housekeeping gene RPN1. No MEL1 fusion transcripts have been identified. MEL1 is not expressed in normal hematopoiesis but in leukemic cells with t(1;3)(p36;q21), and it is believed that RPN1 at 3q21 is the main driver of the ectopic expression of the truncated MEL1 of a protein lacking the PR domain [89–91].

### Clinical and molecular features

The median age of patients with t(1;3)(p36;q21) is 60 years, with a reported range of 30–80 years, and occurrence is typically equal among males and females. Fifteen to 20% of t(1;3) have prior genotoxic exposure [42, 92]. However, in contrast to most other reciprocal translocations in t-AML, t(1;3) is associated with irradiation and alkylating agents rather than with topoisomerase II inhibitors [93–96].

AML with t(1;3)(p32q21) shows cytomorphological characteristics similar to those of AML MECCOM rearrangements with trilinear dysplasia and an excess of small monolobated clustered megakaryocytes, with one-third having high platelet counts [71, 97]. Blast cells show monocytic differentiation with low myeloperoxidase expression [98]. To our knowledge, a characteristic flow cytometry immunophenotypic profile has not been described for this entity.

The t(1,3) aberration is typically identified by conventional karyotyping. Most of the t(1;3)-positive myeloid neoplasms described are AML and are diagnosed during a short MDS pre-phase [99, 100]. In two-thirds of cases, no other cytogenetic alterations are found. The most frequent additional changes are a complex karyotype in approximately 20% and del(5q) found in 15%. The t(1;3) has been described in a small number of cases with acute promyelocytic leukemia and chronic myeloid leukemia [85, 92]. In the Mitelman Database of Chromosome Aberration and Gene Fusions in Cancer, there are now 60 cases of AML and 23 cases of MDS carrying this translocation.

### Response to chemotherapy and prognosis

AML with t(1,3) is associated with non-responsiveness to conventional chemotherapy and a dismal prognosis [85, 93, 97, 99, 101]. In a review of 36 patients with t(1;3)(p32q21 (i.e., including some ALL cases), only a few patients with t(1;3) experienced complete remission, and the median survival was 21.3 months; the authors suggested that stem cell transplant should be offered in the first remission [92].

### Differences between adult and pediatric AML patients; possible relevance for the uncommon abnormalities included in this subclassification

Pediatric and adult AML patients differ with regard to the frequencies of various genetic abnormalities; chromosomal abnormalities are generally more common in pediatric patients and a normal karyotype is thereby less common (20% versus 50%), whereas molecular genetic abnormalities are generally fewer per patient and with only a limited number of AML-associated molecular abnormalities being frequently detected [102]. Some important differences are:

- Cytogenetic abnormalities t(8;21)(p22;q22), inv(16)(p13;q22) and t(16;16)(p13.1;q22) are more prevalent in pediatric AML, and they are regarded as favorable both for adult and pediatric patients. Certain translocations creating fusion genes (e.g. *RUNX1-RUNX1T1*, *KMT2A* rearrangements, *NUP98-NSD1*) as well as certain translocations have a much higher frequency in pediatric AML, e.g. t(1;22)(p13;q13/*RBM15-MKL1*), t(7;12) (q36;p13/*ETV6-MNX1* and t(11;12)/(p15;q13)/*NUP98-KDM5A* [102, 103]. Some other cytogenetic abnormalities associated with adverse prognosis also seem to occur mainly/only in pediatric AML, i.e. t(5;11)(*NUP98/NSD1*) and inv(16)(*CBFA2T3/GLIS2*) that is seen in Down syndrome-associated acute megakaryoblastic leukemia [102].
- Abnormalities involving 11q23 are also more frequent in younger/pediatric patients, whereas complex cytogenetic abnormalities are more frequent in elderly patients [102].
- Relatively frequently mutated genes in pediatric AML include especially *CEBPA* (11%), *WT1* (7.8%) and *ASXL1/2* (8.8%) [104]. On the other hand, other mutation (e.g. DNMT3A that is very rare, NPM1 with 3.8% occurrence) are less common in pediatric AML [102, 104].
- Complex cytogenetic abnormalities have an adverse prognostic impact in both groups although this impact has been regarded as weaker in pediatric than in adult patients [102, 104–107], and monosomal karyotype does not seem to have an adverse impact in pediatric but only adult AML [108].
- The large majority of pediatric patients has de novo AML; secondary AML following previous hematological disease or chemotherapy is much less common in pediatric AML although it has been described for certain abnormalities, e.g. t(16;21(q24;q22) after treatment with topoisomerase 2 II inhibitors [109, 110].
- There are also age-dependent differences within pediatric AML; this is not only true for infant AML that is regarded as a separate entity that include most cases with inv(16)(*CBFA2T3/GLIS2*) and t(7;12)(*MNX1/ETV6*) [102, 111], but also within the group of elderly pediatric patients with regard to for example NPM1 mutations [102].

Certain AML-associated abnormalities can be detected at the time of birth and have probably occurred in utero, e.g. GATA1 [112] and certain MLL-rearrangements [113, 114]. It is not known whether the infant disease with such abnormalities is similar to AML with the same genetic abnormality but occurring in later childhood/adolescence/adults. AML-associated abnormalities present at birth may also be due to germline mutations causing predisposition for AML [115]; such predisposition does not necessarily lead to pediatric AML because at least certain abnormalities (e.g. RUNX1 and DDX41 mutations are associated with AML in adults [115–117]. One should also remember that many patients with germline cancer predisposition lack a clear family history consistent with cancer predisposition [115, 118].

Taken together these observations show that adult and pediatric AML differ with regard to clinical factors (de novo versus secondary) as well as the frequency/occurrence of various leukemia-associated genetic abnormalities. However, even AML-predisposing germline mutations can be detected in adult AML. Several of the abnormalities included in the AML patient subset discussed in this review also differ between pediatric and adult AML, e.g. inv(16) (*CBFA2T3/GLIS2*) and t(7;12) (q36;p13/*ETV6-MNX1* are only/mainly observed in children, t(10;11) is mainly detected in adolescence/young adults whereas other abnormalities are observed in both pediatric and human AML. All these abnormalities are uncommon, and it is not known whether additional signs of aging in the transformed cells modulate the contributions of these abnormalities in leukemogenesis or their prognostic impact in patients receiving antileukemic treatment.

## Conclusion

The recent changes in current classification systems with addition of multiple genetically defined subgroups reflect the growing knowledge of the genetic diversity of AML. AML with rare recurring translocation is a heterogeneous group of AMLs with a wide range of clinical, laboratory and prognostic features. Importantly, diagnostic workout for some of these subtypes is often omitted in adult patients due to their rarity in adult patients. Although new diagnostic tools such as targeted RNA next-generation sequencing would allow for detection of a larger proportion of these translocations in adult patients, larger cooperative work is required to better characterize these entities. The major challenge in the rare AML variants is to incorporate clinical and molecular data to develop robust algorithms for risk stratification, guide consolidation therapy that might allow for exploration of new targeted drugs.

## References

[1] Khoury JD, Solary E, Abla O (2022). The 5th edition of the World Health Organization Classification of Haematolymphoid Tumours: myeloid and Histiocytic/Dendritic neoplasms. Leukemia.

[2] Arber DA, Orazi A, Hasserjian RP (2022). International Consensus classification of myeloid neoplasms and Acute Leukemias: integrating morphologic, clinical, and genomic data. Blood.

[3] Gough SM, Slape CI, Aplan PD (2011). NUP98 gene fusions and hematopoietic malignancies: common themes and new biologic insights. Blood.

[4] Michmerhuizen NL, Klco JM, Mullighan CG (2020). Mechanistic insights and potential therapeutic approaches for NUP98-rearranged hematologic malignancies. Blood.

[5] Hollink IH, van den Heuvel-Eibrink MM, Arentsen-Peters ST (2011). NUP98/NSD1 characterizes a novel poor prognostic group in acute myeloid leukemia with a distinct HOX gene expression pattern. Blood.

[6] Mohanty S (2023). NUP98 rearrangements in AML: Molecular mechanisms and clinical implications. Onco.

[7] Thol F, Kölking B, Hollink IHI (2013). Analysis of NUP98/NSD1 translocations in adult AML and MDS patients. Leukemia.

[8] Xie W, Hu S, Xu J (2019). Acute myeloid leukemia with t(8;16)(p11.2;p13.3)/KAT6A-CREBBP in adults. Ann Hematol.

[9] Quessada J, Cuccuini W, Saultier P (2021). Cytogenetics of Pediatric Acute myeloid leukemia: a review of the current knowledge. Genes.

[10] Marceau-Renaut A, Duployez N, Ducourneau B (2018). Molecular profiling defines distinct prognostic subgroups in Childhood AML: a Report from the French ELAM02 Study Group. HemaSphere.

[11] Bolouri H, Farrar JE, Triche T (2018). The molecular landscape of pediatric acute myeloid leukemia reveals recurrent structural alterations and age-specific mutational interactions. Nat Med.

[12] Struski S, Lagarde S, Bories P (2017). NUP98 is rearranged in 3.8% of pediatric AML forming a clinical and molecular homogenous group with a poor prognosis. Leukemia.

[13] Niktoreh N, Walter C, Zimmermann M (2019). Mutated WT1, FLT3-ITD, and NUP98-NSD1 Fusion in various combinations define a Poor Prognostic Group in Pediatric Acute myeloid leukemia. J Oncol.

[14] Shiba N, Ichikawa H, Taki T (2013). NUP98-NSD1 gene fusion and its related gene expression signature are strongly associated with a poor prognosis in pediatric acute myeloid leukemia. Genes Chromosomes Cancer.

[15] Bertrums EJM, Smith JL, Harmon L (2023). Comprehensive molecular and clinical characterization of NUP98 fusions in pediatric acute myeloid leukemia. Haematologica.

[16] Romana SP, Radford-Weiss I, Ben Abdelali R (2006). NUP98 rearrangements in hematopoietic malignancies: a study of the Groupe Francophone De Cytogénétique Hématologique. Leukemia.

[17] Jaju RJ, Haas OA, Neat M (1999). A new recurrent translocation, t(5;11)(q35;p15.5), associated with Del(5q) in childhood acute myeloid leukemia. The UK Cancer Cytogenetics Group (UKCCG). Blood.

[18] Hara Y, Shiba N, Ohki K (2017). Prognostic impact of specific molecular profiles in pediatric acute megakaryoblastic leukemia in non-down syndrome. Genes Chromosom Cancer.

[19] de Rooij JDE, Hollink IHIM, Arentsen-Peters STCJM (2013). NUP98/JARID1A is a novel recurrent abnormality in pediatric acute megakaryoblastic leukemia with a distinct HOX gene expression pattern. Leukemia.

[20] Tao S, Song L, Deng Y (2020). Acute myeloid leukemia with NUP98-RARG Gene Fusion similar to Acute Promyelocytic Leukemia: Case Report and Literature Review. Onco Targets Ther.

[21] Zhu HH, Yang MC, Wang F (2020). Identification of a novel NUP98-RARA fusion transcript as the 14th variant of acute promyelocytic leukemia. Am J Hematol.

[22] Ostronoff F, Othus M, Gerbing RB (2014). NUP98/NSD1 and FLT3/ITD coexpression is more prevalent in younger AML patients and leads to induction failure: a COG and SWOG report. Blood.

[23] de Rooij JD, Hollink IH, Arentsen-Peters ST (2013). NUP98/JARID1A is a novel recurrent abnormality in pediatric acute megakaryoblastic leukemia with a distinct HOX gene expression pattern. Leukemia.

[24] Hara Y, Shiba N, Ohki K (2017). Prognostic impact of specific molecular profiles in pediatric acute megakaryoblastic leukemia in non-down syndrome. Genes Chromosomes Cancer.

[25] Gruber TA, Larson Gedman A, Zhang J (2012). An inv(16)(p13.3q24.3)-encoded CBFA2T3-GLIS2 fusion protein defines an aggressive subtype of pediatric acute megakaryoblastic leukemia. Cancer Cell.

[26] Masetti R, Bertuccio SN, Pession A, Locatelli F (2019). CBFA2T3-GLIS2-positive acute myeloid leukaemia. A peculiar paediatric entity. Br J Haematol.

[27] Noort S, Zimmermann M, Reinhardt D (2018). Prognostic impact of t(16;21)(p11;q22) and t(16;21)(q24;q22) in pediatric AML: a retrospective study by the I-BFM Study Group. Blood.

[28] Zerkalenkova E, Panfyorova A, Kazakova A (2018). Molecular characteristic of acute leukemias with t(16;21)/FUS-ERG. Ann Hematol.

[29] Eidenschink Brodersen L, Alonzo TA, Menssen AJ (2016). A recurrent immunophenotype at diagnosis independently identifies high-risk pediatric acute myeloid leukemia: a report from children’s Oncology Group. Leukemia.

[30] Liu H, Wang SA, Schlette EJ (2018). Myeloid neoplasms with t(16;21)(q24;q22)/RUNX1-RUNX1T3 mimics acute myeloid leukemia with RUNX1-RUNX1T1. Ann Hematol.

[31] Kitabayashi I, Aikawa Y, Nguyen LA (2001). Activation of AML1-mediated transcription by MOZ and inhibition by the MOZ-CBP fusion protein. EMBO J.

[32] Yang XJ, Seto E (2007). HATs and HDACs: from structure, function and regulation to novel strategies for therapy and prevention. Oncogene.

[33] Chan EM, Chan RJ, Comer EM (2007). MOZ and MOZ-CBP cooperate with NF-kappaB to activate transcription from NF-kappaB-dependent promoters. Exp Hematol.

[34] Camos M, Esteve J, Jares P (2006). Gene expression profiling of acute myeloid leukemia with translocation t(8;16)(p11;p13) and MYST3-CREBBP rearrangement reveals a distinctive signature with a specific pattern of HOX gene expression. Cancer Res.

[35] Diab A, Zickl L, Abdel-Wahab O (2013). Acute myeloid leukemia with translocation t(8;16) presents with features which mimic acute promyelocytic leukemia and is associated with poor prognosis. Leuk Res.

[36] Vo N, Goodman RH (2001). CREB-binding protein and p300 in transcriptional regulation. J Biol Chem.

[37] Blobel GA (2000). CREB-binding protein and p300: molecular integrators of hematopoietic transcription. Blood.

[38] Chen J, Odenike O, Rowley JD (2010). Leukaemogenesis: more than mutant genes. Nat Rev Cancer.

[39] Gervais C, Murati A, Helias C (2008). Acute myeloid leukaemia with 8p11 (MYST3) rearrangement: an integrated cytologic, cytogenetic and molecular study by the groupe francophone de cytogenetique hematologique. Leukemia.

[40] Haferlach T, Kohlmann A, Klein HU (2009). AML with translocation t(8;16)(p11;p13) demonstrates unique cytomorphological, cytogenetic, molecular and prognostic features. Leukemia.

[41] Hanslip JI, Swansbury GJ, Pinkerton R, Catovsky D The translocation t(8;16)(p11;p13) defines an AML subtype with distinct cytology and clinical features. Leukemia & Lymphoma. 1992 1992/01/01;6(6):479–486

[42] Heim S, Mitelman F, Chichester (2015) West Sussex: John Wiley & Sons, Inc. Chichester, West Sussex

[43] Kayser S, Hills RK, Langova R (2021). Characteristics and outcome of patients with acute myeloid leukaemia and t(8;16)(p11;p13): results from an International Collaborative Study. Br J Haematol.

[44] Coenen EA, Zwaan CM, Reinhardt D (2013). Pediatric acute myeloid leukemia with t(8;16)(p11;p13), a distinct clinical and biological entity: a collaborative study by the International-Berlin-Frankfurt-Munster AML-study group. Blood.

[45] Borel C, Huynh A, Chaufour X (2010). Uterine chloroma, aortic thrombus and CALM/AF10 acute myeloid leukemia. Leuk Res.

[46] Caudell D, Aplan PD (2008). The role of CALM-AF10 gene fusion in acute leukemia. Leukemia.

[47] Archangelo LF, Glasner J, Krause A, Bohlander SK (2006). The novel CALM interactor CATS influences the subcellular localization of the leukemogenic fusion protein CALM/AF10. Oncogene.

[48] Dik WA, Brahim W, Braun C (2005). CALM-AF10 + T-ALL expression profiles are characterized by overexpression of HOXA and BMI1 oncogenes. Leukemia.

[49] DiNardo CD, Tang G, Pemmaraju N (2015). Acute myeloid leukemia with t(10;11): a pathological entity with distinct clinical presentation. Clin Lymphoma Myeloma Leuk.

[50] Mark C, Kolb EA, Goemans BF (2022). Treatment outcomes of Childhood Picalm:MLLT10 + Acute Leukemias: An International Retrospective Study. Blood.

[51] Grimwade D, Hills RK, Moorman AV (2010). Refinement of cytogenetic classification in acute myeloid leukemia: determination of prognostic significance of rare recurring chromosomal abnormalities among 5876 younger adult patients treated in the United Kingdom Medical Research Council trials. Blood.

[52] Bacher U, Kern W, Schnittger S (2005). Population-based age-specific incidences of cytogenetic subgroups of acute myeloid leukemia. Haematologica.

[53] Savage NM, Kota V, Manaloor EJ (2010). Acute leukemia with PICALM-MLLT10 fusion gene: diagnostic and treatment struggle. Cancer Genet Cytogenet.

[54] Park M-S, Kim H-Y, Lee JJ (2023). The First Case of Acute myeloid leukemia with t(10;11)(p13;q21); PICALM-MLLT10 rearrangement presenting with extensive skin involvement. Annals Lab Med.

[55] Borel C, Dastugue N, Cances-Lauwers V (2012). PICALM-MLLT10 acute myeloid leukemia: a French cohort of 18 patients. Leuk Res.

[56] La Starza R, Crescenzi B, Krause A (2006). Dual-color split signal fluorescence in situ hybridization assays for the detection of CALM/AF10 in t(10;11)(p13;q14-q21)-positive acute leukemia. Haematologica.

[57] Dreyling MH, Schrader K, Fonatsch C (1998). MLL and CALM are fused to AF10 in morphologically distinct subsets of acute leukemia with translocation t(10;11): both rearrangements are associated with a poor prognosis. Blood.

[58] Grossmann V, Bacher U, Kohlmann A (2012). EZH2 mutations and their association with PICALM-MLLT10 positive acute leukaemia. Br J Haematol.

[59] Ou DM, Liu GX, Yan JY (2004). [CALM-AF10 fusion transcripts in primary leukemia with t(10;11) and in vitro chemotherapy sensitivity of leukemic cells with t(10;11)]. Zhongguo Shi Yan Xue Ye Xue Za Zhi.

[60] Sarkaria SM, Christopher MJ, Klco JM, Ley TJ (2014). Primary acute myeloid leukemia cells with IDH1 or IDH2 mutations respond to a DOT1L inhibitor in vitro. Leukemia.

[61] Carroll A, Civin C, Schneider N (1991). The t(1;22) (p13;q13) is nonrandom and restricted to infants with acute megakaryoblastic leukemia: a Pediatric Oncology Group Study. Blood.

[62] Mercher T, Raffel GD, Moore SA (2009). The OTT-MAL fusion oncogene activates RBPJ-mediated transcription and induces acute megakaryoblastic leukemia in a knockin mouse model. J Clin Invest.

[63] Chisholm KM, Smith J, Heerema-McKenney AE (2023). Pathologic, cytogenetic, and molecular features of acute myeloid leukemia with megakaryocytic differentiation: a report from the Children’s Oncology Group. Pediatr Blood Cancer.

[64] Saito Y, Makita S, Chinen S (2020). Acute megakaryoblastic leukaemia with t(1;22)(p13·3;q13·1)/RBM15-MKL1 in an adult patient following a non-mediastinal germ cell tumour. Br J Haematol.

[65] Hsiao H-H, Yang M-Y, Liu Y-C (2005). RBM15-MKL1 (OTT-MAL) fusion transcript in an adult acute myeloid leukemia patient. Am J Hematol.

[66] de Rooij JD, Masetti R, van den Heuvel-Eibrink MM (2016). Recurrent abnormalities can be used for risk group stratification in pediatric AMKL: a retrospective intergroup study. Blood.

[67] Lion T, Haas OA, Harbott J (1992). The translocation t(1;22)(p13;q13) is a nonrandom marker specifically associated with acute megakaryocytic leukemia in young children. Blood.

[68] Dastugue N, Lafage-Pochitaloff M, Pagès M-P (2002). Cytogenetic profile of childhood and adult megakaryoblastic leukemia (M7): a study of the Groupe Français De Cytogénétique Hématologique (GFCH). Blood.

[69] Marques-Piubelli ML, Cordeiro MG, Cristofani L (2020). Acute megakaryoblastic leukemia with t(1;22)(p13.3;q13.1); RBM15-MKL1 mimicking hepatoblastoma in an infant: the role of karyotype in differential diagnosis. Pediatr Blood Cancer.

[70] Brouwer N, Matarraz S, Nierkens S et al (2022) Immunophenotypic analysis of Acute Megakaryoblastic Leukemia: a EuroFlow Study. Cancers (Basel). ;14(6) 10.3390/cancers14061583 PMC8946548 35326734

[71] Lim G, Choi JR, Kim MJ (2010). Detection of t(3;5) and NPM1/MLF1 rearrangement in an elderly patient with acute myeloid leukemia: clinical and laboratory study with review of the literature. Cancer Genet Cytogenet.

[72] Dumézy F, Renneville A, Mayeur-Rousse C (2013). Acute myeloid leukemia with translocation t(3;5): new molecular insights. Haematologica.

[73] Aypar U, Knudson RA, Pearce KE (2014). Development of an NPM1/MLF1 D-FISH probe set for the detection of t(3;5)(q25;q35) identified in patients with acute myeloid leukemia. J Mol Diagn.

[74] Alattar ML, Kantarjian HM, Cortes JE (2012). Incidence and outcomes of a rare translocation t(3,5) in patients (pts) with Acute myeloid leukemia (AML) and myelodysplastic syndrome (MDS). Blood.

[75] Tosi S, Harbott J, Teigler-Schlegel A (2000). T(7;12)(q36;p13), a new recurrent translocation involving ETV6 in infant leukemia. Genes Chromosomes Cancer.

[76] Park J, Kim M, Lim J (2009). Three-way complex translocations in infant acute myeloid leukemia with t(7;12)(q36;p13): the incidence and correlation of a HLXB9 overexpression. Cancer Genet Cytogenet.

[77] von Bergh AR, van Drunen E, van Wering ER (2006). High incidence of t(7;12)(q36;p13) in infant AML but not in infant ALL, with a dismal outcome and ectopic expression of HLXB9. Genes Chromosomes Cancer.

[78] Ballabio E, Cantarella CD, Federico C (2009). Ectopic expression of the HLXB9 gene is associated with an altered nuclear position in t(7;12) leukaemias. Leukemia.

[79] Ragusa D, Dijkhuis L, Pina C, Tosi S (2023) Mechanisms associated with t(7;12) acute myeloid leukaemia: from genetics to potential treatment targets. Biosci Rep. ;43(1) 10.1042/BSR20220489 PMC9894016 36622782

[80] Waraky A, Östlund A, Arabanian L (2019). The translocation t(7;12)(q36;p13) induces myeloid leukemia in Immuno-compromised but not immunocompetent mice. Blood.

[81] Slater RM, von Drunen E, Kroes WG (2001). T(7;12)(q36;p13) and t(7;12)(q32;p13)--translocations involving ETV6 in children 18 months of age or younger with myeloid disorders. Leukemia.

[82] Espersen ADL, Noren-Nyström U, Abrahamsson J (2018). Acute myeloid leukemia (AML) with t(7;12)(q36;p13) is associated with infancy and trisomy 19: data from Nordic Society for Pediatric Hematology and Oncology (NOPHO-AML) and review of the literature. Genes Chromosomes Cancer.

[83] Taketani T, Taki T, Sako M (2008). MNX1-ETV6 fusion gene in an acute megakaryoblastic leukemia and expression of the MNX1 gene in leukemia and normal B cell lines. Cancer Genet Cytogenet.

[84] Shimizu S, Suzukawa K, Kodera T (2000). Identification of breakpoint cluster regions at 1p36.3 and 3q21 in hematologic malignancies with t(1;3)(p36;q21). Genes Chromosomes Cancer.

[85] Sato Y, Murai M, Tsunoda J (1991). Second relapse of acute promyelocytic leukemia (ANLL-M3) with t(15;17) and t(1;3)(p36;q21). Cancer Genet Cytogenet.

[86] Milton JM, Garson OM, Paton CM, Hurley TF (1984). A complicated but nonrandom karyotype in preleukemia. Cancer Genet Cytogenet.

[87] Swerdlow SH, Campo E, Pileri SA (2016). The 2016 revision of the World Health Organization classification of lymphoid neoplasms. Blood.

[88] Mochizuki N, Shimizu S, Nagasawa T (2000). A novel gene, MEL1, mapped to 1p36.3 is highly homologous to the MDS1/EVI1 gene and is transcriptionally activated in t(1;3)(p36;q21)-positive leukemia cells. Blood.

[89] Lahortiga I, Agirre X, Belloni E (2004). Molecular characterization of a t(1;3)(p36;q21) in a patient with MDS. MEL1 is widely expressed in normal tissues, including bone marrow, and it is not overexpressed in the t(1;3) cells. Oncogene.

[90] Nishikata I, Sasaki H, Iga M (2003). A novel EVI1 gene family, MEL1, lacking a PR domain (MEL1S) is expressed mainly in t(1;3)(p36;q21)-positive AML and blocks G-CSF-induced myeloid differentiation. Blood.

[91] Xiao Z, Zhang M, Liu X (2006). MEL1S, not MEL1, is overexpressed in myelodysplastic syndromes patients with t(1;3)(p36;q21). Leuk Res.

[92] Lim G, Kim MJ, Oh SH (2010). Acute myeloid leukemia associated with t(1;3)(p36;q21) and extreme thrombocytosis: a clinical study with literature review. Cancer Genet Cytogenet.

[93] Welborn JL, Lewis JP, Jenks H, Walling P (1987). Diagnostic and prognostic significance of t(1;3)(p36;q21) in the disorders of hematopoiesis. Cancer Genet Cytogenet.

[94] Block, Carroll, Hagemeijer (2002). Rare recurring balanced chromosome abnormalities in therapy-related myelodysplastic syndromes and acute leukemia: report from an international workshop. Genes Chromosomes Cancer.

[95] Charrin C, Belhabri A, Treille-Ritouet D (2002). Structural rearrangements of chromosome 3 in 57 patients with acute myeloid leukemia: clinical, hematological and cytogenetic features. Hematol J.

[96] Mauritzson N, Albin M, Rylander L (2002). Pooled analysis of clinical and cytogenetic features in treatment-related and de novo adult acute myeloid leukemia and myelodysplastic syndromes based on a consecutive series of 761 patients analyzed 1976–1993 and on 5098 unselected cases reported in the literature 1974–2001. Leukemia.

[97] Duhoux FP, Ameye G, Montano-Almendras CP (2012). PRDM16 (1p36) translocations define a distinct entity of myeloid malignancies with poor prognosis but may also occur in lymphoid malignancies. Br J Haematol.

[98] Eveillard M, Delaunay J, Richebourg S (2015). The closely related rare and severe acute myeloid leukemias carrying EVI1 or PRDM16 rearrangements share singular biological features. Haematologica.

[99] Moir DJ, Jones PA, Pearson J (1984). A new translocation, t(1;3) (p36;q21), in myelodysplastic disorders. Blood.

[100] Secker-Walker LM, Mehta A, Bain B (1995). Abnormalities of 3q21 and 3q26 in myeloid malignancy: a United Kingdom Cancer Cytogenetic Group study. Br J Haematol.

[101] Bloomfield CD, Garson OM, Volin L (1985). T(1;3)(p36;q21) in acute nonlymphocytic leukemia: a new cytogenetic-clinicopathologic association. Blood.

[102] Chaudhury SS, Morison JK, Gibson BE, Keeshan K (2015). Insights into cell ontogeny, age, and acute myeloid leukemia. Exp Hematol.

[103] de Rooij JD, Branstetter C, Ma J (2017). Pediatric non-down syndrome acute megakaryoblastic leukemia is characterized by distinct genomic subsets with varying outcomes. Nat Genet.

[104] Shiba N, Yoshida K, Shiraishi Y (2016). Whole-exome sequencing reveals the spectrum of gene mutations and the clonal evolution patterns in paediatric acute myeloid leukaemia. Br J Haematol.

[105] Creutzig U, van den Heuvel-Eibrink MM, Gibson B (2012). Diagnosis and management of acute myeloid leukemia in children and adolescents: recommendations from an international expert panel. Blood.

[106] Niewerth D, Creutzig U, Bierings MB, Kaspers GJ (2010). A review on allogeneic stem cell transplantation for newly diagnosed pediatric acute myeloid leukemia. Blood.

[107] Tarlock K, Sulis ML, Chewning JH et al (2022) Hematopoietic cell transplantation in the treatment of Pediatric Acute Myelogenous Leukemia and Myelodysplastic syndromes: guidelines from the American Society of Transplantation and Cellular Therapy. Transplantation and Cellular Therapy, Official Publication of the American Society for Transplantation and Cellular Therapy. 28(9):530–545 10.1016/j.jtct.2022.06.005 35717004

[108] Lee NH, Choi YB, Yi ES (2016). Monosomal karyotype is not a predictor of dismal outcome in childhood de novo acute myeloid leukemia. Leuk Res.

[109] Boils CL, Mohamed AN (2008). T(16;21)(q24;q22) in acute myeloid leukemia: case report and review of the literature. Acta Haematol.

[110] Crisci S, Pota E, Iaccarino G (2020). Childhood therapy-related Acute myeloid leukemia with t(16;21)(q24;q22)/RUNX1-CBFA2T3 after a primitive neuroectodermal tumor of the Chest Wall. Clin Lymphoma Myeloma Leuk.

[111] Calvo C, Fenneteau O, Leverger G et al (2021) Infant Acute myeloid leukemia: a Unique Clinical and Biological Entity. Cancers (Basel). ;13(4) 10.3390/cancers13040777 PMC7918235 33668444

[112] Pine SR, Guo Q, Yin C (2007). Incidence and clinical implications of GATA1 mutations in newborns with Down syndrome. Blood.

[113] Ford AM, Ridge SA, Cabrera ME (1993). In utero rearrangements in the trithorax-related oncogene in infant leukaemias. Nature.

[114] Gale KB, Ford AM, Repp R (1997). Backtracking leukemia to birth: identification of clonotypic gene fusion sequences in neonatal blood spots. Proc Natl Acad Sci U S A.

[115] Klco JM, Mullighan CG (2021). Advances in germline predisposition to acute leukaemias and myeloid neoplasms. Nat Rev Cancer.

[116] Antony-Debré I, Duployez N, Bucci M (2016). Somatic mutations associated with leukemic progression of familial platelet disorder with predisposition to acute myeloid leukemia. Leukemia.

[117] Sébert M, Passet M, Raimbault A (2019). Germline DDX41 mutations define a significant entity within adult MDS/AML patients. Blood.

[118] Zhang J, Walsh MF, Wu G (2015). Germline mutations in predisposition genes in Pediatric Cancer. N Engl J Med.

[119] Wu A, Liu Y, Wei S (2023). Clinical features of patients with acute myeloid leukaemia and the NUP98::NSD1 fusion gene. Int J Lab Hematol.

[120] Noort S, Wander P, Alonzo TA (2021). The clinical and biological characteristics of NUP98-KDM5A in pediatric acute myeloid leukemia. Haematologica.

[121] De Braekeleer E, Douet-Guilbert N, Morel F (2011). RUNX1 translocations and fusion genes in malignant hemopathies. Future Oncol.

[122] Masetti R, Pigazzi M, Togni M (2013). CBFA2T3-GLIS2 fusion transcript is a novel common feature in pediatric, cytogenetically normal AML, not restricted to FAB M7 subtype. Blood.

[123] Masetti R, Rondelli R, Fagioli F (2014). Infants with acute myeloid leukemia treated according to the Associazione Italiana Di Ematologia E Oncologia Pediatrica 2002/01 protocol have an outcome comparable to that of older children. Haematologica.

[124] Park IJ, Park JE, Kim HJ (2010). Acute myeloid leukemia with t(16;21)(q24;q22) and eosinophilia: case report and review of the literature. Cancer Genet Cytogenet.

[125] Toda Y, Nagai Y, Shimomura D (2017). Acute basophilic leukemia associated with the t(16;21)(p11;q22)/FUS-ERG fusion gene. Clin Case Rep.

[126] Saucedo-Campos A, Islas-Perez A, Lopez-Martinez B (2020). Acute myeloid leukemia associated with t(16:21)(p11;q22) in a pediatric patient. Bol Med Hosp Infant Mex.

[127] Shikami M, Miwa H, Nishii K (1999). Myeloid differentiation antigen and cytokine receptor expression on acute myelocytic leukaemia cells with t(16;21)(p11;q22): frequent expression of CD56 and interleukin-2 receptor alpha chain. Br J Haematol.

